# Parthenolide and costunolide reduce microtentacles and tumor cell attachment by selectively targeting detyrosinated tubulin independent from NF-κB inhibition

**DOI:** 10.1186/bcr3477

**Published:** 2013-09-13

**Authors:** Rebecca A Whipple, Michele I Vitolo, Amanda E Boggs, Monica S Charpentier, Keyata Thompson, Stuart S Martin

**Affiliations:** 1 Marlene and Stewart Greenebaum NCI Cancer Center, University of Maryland School of Medicine, Bressler Bldg. Rm 10-29, 22 S. Greene Street, Baltimore, MD 21201, USA; 2 Department of Physiology, University of Maryland School of Medicine, Bressler Bldg. Rm 10-29, 655 W. Baltimore Street, Baltimore, MD 21201, USA; 3 Program in Molecular Medicine, University of Maryland School of Medicine, Bressler Bldg. Rm 10-29, 655 W. Baltimore Street, Baltimore, MD 21201, USA

## Abstract

**Introduction:**

Detyrosinated tubulin, a post-translational modification of α-tubulin and a hallmark of stable microtubules, has gained recent attention given its association with tumor progression, invasiveness, and chemoresistance. We also recently reported that epithelial-to-mesenchymal transition (EMT) promotes tubulin detyrosination through tubulin tyrosine ligase (TTL) suppression. Furthermore, detyrosinated tubulin-enriched membrane protrusions, termed microtentacles (McTN), facilitate tumor cell reattachment to endothelial layers. Given the induction of EMT associated with inflammation and cancer progression, we tested anti-inflammatory nuclear factor-kappaB (NF-κB) inhibitors on a panel of human breast carcinoma cells to examine their effects on detyrosinated tubulin to identify more specific tubulin-directed anti-cancer treatments.

**Methods:**

Using metastatic human breast carcinoma cells MDA-MB-157, MDA-MB-436, and Bt-549, we measured the impact of NF-κB inhibitors parthenolide, costunolide, and resveratrol on detyrosinated tubulin using protein expression analysis and immunofluorescence. A luciferase reporter assay and a viability screen were performed to determine if the effects were associated with their NF-κB inhibitory properties or were a result of apoptosis. Real-time monitoring of cell-substratum attachment was measured utilizing electrical impedance across microelectronic sensor arrays. We compared the selectivity of the NF-κB inhibitors to specifically target detyrosinated tubulin with traditional tubulin-targeted therapeutics, paclitaxel and colchicine, throughout the study.

**Results:**

Sesquiterpene lactones, parthenolide and costunolide, selectively decrease detyrosinated tubulin independent of their inhibition of NF-κB. Live-cell scoring of suspended cells treated with parthenolide and costunolide show reduction in the frequency of microtentacles and inhibition of reattachment. Structural analysis shows that parthenolide and costunolide can decrease detyrosinated microtubules without significantly disrupting the overall microtubule network or cell viability. Paclitaxel and colchicine display indiscriminate disruption of the microtubule network.

**Conclusions:**

Our data demonstrate that selective targeting of detyrosinated tubulin with parthenolide and costunolide can reduce McTN frequency and inhibit tumor cell reattachment. These actions are independent of their effects on NF-κB inhibition presenting a novel anti-cancer property and therapeutic opportunity to selectively target a stable subset of microtubules in circulating tumor cells to reduce metastatic potential with less toxicity in breast cancer patients.

## Introduction

Microtubules have long been a target for cancer therapy given their critical and diverse cellular functions in intracellular transport and metabolism, as well as cell shape, signaling, migration, polarization and division [1]. Despite the attractiveness of microtubules as cancer targets, the clinical effectiveness and tolerance of microtubule-directed agents are limited, largely due to toxicity, broad and/or undetermined mechanisms of action, inherent or acquired resistance and tubulin mutations that reduce drug binding [2]. Alterations in tubulin-binding sites, microtubule associated proteins (MAPs) and microtubule dynamics have all been implicated as mechanisms for tumorigenesis, chemoresistance and metastasis [3]. To improve and advance the therapeutic benefits of microtubule-targeted compounds for cancer treatment, research has focused on combination therapy, discovering novel tubulin or tubulin-associated drug targets and elucidating more specific drug mechanisms. More importantly, better characterization of microtubule function, regulation and role in cancer progression and chemoresistance continue to advance the development of clinically-applicable, novel tubulin-directed chemotherapies.

Microtubules can undergo phases of growth and shrinkage by modulating dynamic instability; however, stabilization of a subset of microtubules is necessary for cell motility [4] and morphogenetic events [5]. Selective stabilization occurs prior to changes in cell behavior [6], indicating that stabilization is an early causative event rather than a result of alterations in cell behavior. These stable microtubules are enriched in detyrosinated tubulin, a reversible post-translational modification on the C-terminus of α-tubulin, regulated by the enzymatic activity of tubulin tyrosine ligase (TTL) and an ill-defined tubulin carboxypeptidase (TCP). Detyrosination has been shown to be a consequence of microtubule stability and the precise function of this post-translational modification on microtubule dynamics and regulation is still unclear. Early insight into microtubule stability suggests that stable microtubules enriched in detyrosinated tubulin are more resistant to microtubule antagonists [4]. In addition to its implications for chemoresistance, research has shown that increased detyrosinated tubulin is associated with poor cancer prognosis [7] and may arise from suppressed TTL activity during tumor growth which prevents re-tyrosination [8,9]. Moreover, TTL-/- cells exhibit decreased microtubule sensitivity to depolymerizing drugs as well as microtubule overgrowth and persistence at the cell’s leading edge [10], potentially contributing to abnormal cell behavior. We have recently reported that epithelial-to-mesenchymal transition (EMT) promotes α-tubulin detyrosination by downregulating expression of TTL and that detyrosinated tubulin accumulates at invasive tumor fronts in patient samples [11]. Furthermore, we have shown that microtentacles (McTNs), tubulin-based, dynamic membrane protrusions that occur at high frequencies in detached metastatic cell lines, are enriched in detyrosinated tubulin and facilitate tumor cell reattachment and cell-cell adhesion [11,12]. This evidence highlights the importance of the tubulin tyrosination cycle in cancer progression and reveals detyrosinated tubulin as a novel microtubule target in the metastatic cascade.

The relationship between EMT, inflammation and cancer progression has received considerable attention in the last several years [13]. Given our recent discovery that EMT promotes detyrosination of α-tubulin in combination with data linking chronic inflammation and associated nuclear factor-kappaB (NF-κB) activation to the induction of an EMT [14,15], we decided to test anti-inflammatory compounds to determine their impact on tubulin detyrosination and McTN occurrence. Parthenolide and costunolide, members of the natural compound sesquiterpene lactone group, have been well-characterized as inhibitors of the NF-κB pathway and effective anti-inflammatory drugs but are less recognized for their microtubule-interfering properties [16,17]. A recent report, however, uncovered parthenolide’s ability to inhibit TCP activity to restore functional tyrosinated tubulin levels and reduce detyrosination [18]. Parthenolide and costunolide could possibly interfere with microtubules through their potent NF-κB inhibitory properties. There is conflicting evidence suggesting that depolymerization of microtubules can decrease translocation of active NF-κB into the nucleus or induce NF-κB activity and transactivation of NF-κB-dependent genes [19,20]. Additionally, microtubule stabilizers such as Taxol may promote NF-κB activation [21] or have no effect [20]. Despite the conflicting data, it is apparent that interfering with microtubule dynamics can affect transcription factor activity. Therefore, the question remains whether the ability of parthenolide and costunolide to reduce stable, detyrosinated tubulin in metastatic breast cancer cells occurs through a mechanism that is dependent or independent of their NF-κB inhibitory properties.

Our past research has used broad microtubule and actin disrupting agents as tools to determine the structure and function of McTNs. These tools have been useful to define McTNs as tubulin-based and distinguish McTNs from actin-based membrane structures, but have limited value when translated to the clinic due to their toxicity and severe disruption of the entire microtubule array. The primary focus of the current report is to identify novel therapeutic candidates that move beyond indiscriminate targeting of microtubules, enabling reductions in McTNs and the reattachment proficiencies of metastatic breast cancer cell lines by specifically targeting detyrosinated tubulin. We also investigate whether the effects of parthenolide and costunolide on detyrosination are related to their NF-κB inhibitory activity or if a separate mechanism is responsible and reveals multi-faceted targets for these anti-cancer therapies.

## Methods

### Cell culture and chemical compounds

Bt-549 and MDA-MB-436 were maintained at 37°C in (D)MEM (Mediatech, Inc., Manassas, VA) in 5% CO_2_ while MDA-MB-157 was cultured in L-15 media (Life Technologies, Carlsbad, CA) at 37°C without CO_2_. Cells were obtained by American Type Culture Collection (Manassas, VA, USA) and growth media was supplemented with 10% fetal bovine serum (FBS) and penicillin-streptomycin (100 μg/ml). Parthenolide (Parth), resveratrol (ResV), colchicine (Col), and paclitaxel (Taxol;Tax) were obtained from Sigma (St. Louis, MO, USA). Costunolide (Cost) was purchased from Chromadex (Santa Ana, CA, USA). Recombinant human TNF-α was from PeproTech (Rocky Hill, NJ, USA). This study did not require approval from an ethics committee.

### Immunoblot

Cells were treated for six hours in growth media using a concentration range containing vehicle (0.1% dimethyl sulfoxide (DMSO)), Parth, Cost, ResV, Col or Tax. Cells were harvested as previously described [12]. Total protein (12 μg) was separated by SDS-PAGE on 4% to 12% NuPage MES Bis-Tris gels (Life Technologies). Membranes were blocked in 5% milk/Tris-buffered saline - Tween (TBST) for one hour at room temperature followed by an overnight incubation at 4°C in polyclonal detyrosinated α-tubulin (1:1000; abCam, Cambridge, MA) poly (ADP-ribose) polymerase (PARP; H-250, Santa Cruz Biotechnology; 1:1000), or monoclonal α-tubulin DM1A (1:5000; Sigma) in 2.5% milk/TBST. Secondary antibodies to immunoglobulin G-horse radish peroxidase (IgG-HRP) were used (1:10000; Jackson ImmunoResearch, West Grove, PA). Densitometry was performed using ImageJ.

### NF-κB activation

Cells were transduced with NF-κB -luciferase reporter adenovirus (Ad-NF-κB -Luc; 1 × 10^6^ PFU/ml) obtained from Vector Biolabs (Philadelphia, PA, USA) for 24 hours. Cells were then split into a microplate to ensure uniform cell density. Following four hours of drug treatment, cells were stimulated with TNF-α (100 ng/ml) for one hour remaining in the presence of drug. D-Luciferin (Caliper Life Sciences, Alameda, CA; 200 μg/ml) was added and luminescence was detected on a Berthold LB 940 Mirthras. All values are shown as mean ± SD of triplicate samples.

### Cell viability

Cells were seeded into 96-well microplates and treated in triplicate for six hours. CellTiter 96 AQ_ueous_ One Solution (Promega, Madison, WI) was followed according to the manufacturer’s protocol to determine cell viability. Absorbance was measured using a Biotek Synergy HT Multidetection Microplate Reader. All values are shown as mean ± SD of triplicate samples.

### Live cell imaging and microtentacle scoring

GFP-membrane targeted AcGFP1-Mem plasmid (Clontech, Mountain View, CA) was used to generate a custom adenovirus (Ad-GFP-Mem; Vector BioLabs) for McTN scoring and population imaging. Cells were transduced for 24 hours and then treated with drug for 6 hours prior to detachment. Detailed methods for live cell imaging and McTN scoring were previously described [12]. Single Ad-GFP-Mem+cells were scored blindly for McTNs at 15 to 30 minutes while suspended in an ultra-low attachment plate (Corning, Corning, NY) in the respective drug-containing growth media. Images were collected using an Olympus CKX41 inverted fluorescent microscope (Melville, NY, USA) and analysis was performed using Olympus MicroSuite Five software. Cell images were collected following detachment as grayscale images and subsequently color inverted for better visualization contrast. The original images were contrasted equivalently using ImageJ.

### Indirect immunofluorescence

Cells were drug treated on glass coverslips for six hours then fixed in 3.7% formaldehyde/PBS. Fixed cells were permeabilized (0.25% Triton X-100/PBS, 10 minutes), and blocked for one hour (PBS/5% bovine serum albumin (BSA)/0.5% NP40). Immunostaining was performed overnight at 4°C (PBS/2% BSA/0.5% NP40) using polyclonal detyrosinated α-tubulin (abCam) and monoclonal α-tubulin DM1A (Sigma). Image acquisition was captured on an Olympus FV1000 laser scanning confocal microscope (Olympus, Center Valley, PA, USA).

### Cell-electrode impedance attachment assay

Real-time monitoring of cell-substratum attachment was measured utilizing the xCELLigence RTCA SP real-time cell sensing device (ACEA Biosciences, San Diego, CA). Cells were pretreated for six hours, trypsinized and counted. Cells (20,000) were seeded into 96-well microelectronic sensored standard plates (E-plates) containing the respective drug. Briefly, attachment is measured from the interaction of cells with the electrodes and represented as a change in cell index (CI), an arbitrary unit derived from the relative change in electrical impedance across microelectronic sensor arrays. The electrical impedance was captured every three minutes for an experimental duration of 3.5 hours. The attachment rate is expressed as the CI, or the change in electrical impedance at each timepoint. Values are expressed as the +/- SD of the triplicate wells. Three independent trials were conducted.

## Results

### Parthenolide and costunolide decrease detyrosinated tubulin in human breast carcinoma cells

**Figure 1:**
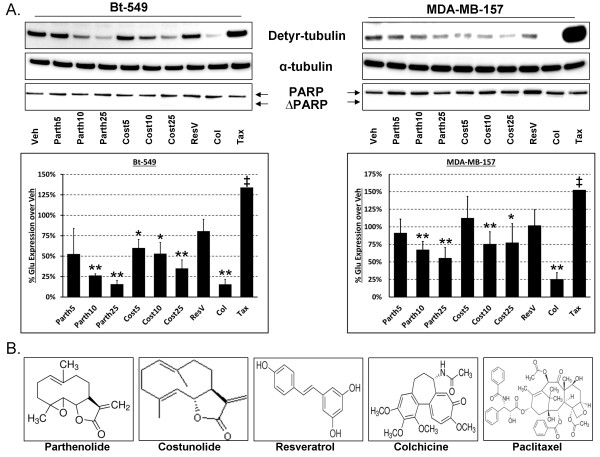
**Parthenolide and costunolide decrease detyrosinated tubulin in human breast carcinoma cells. (A)** Bt‒549 (N = 3) and MDA‒MB‒157 (N = 6) cells treated for six hours with DMSO (Veh; 0.1%), parthenolide (Parth; 5 μM, 10 μM, 25 μM), costunolide (Cost; 5 μM, 10 μM, 25 μM), resveratrol (ResV; 50 μg/ml), colchicine (Col; 50 μM), and Taxol (Tax; 0.5 μg/ml). Parthenolide (10 μM, 25 μM), costunolide (10 μM, 25 μM), and colchicine (50 μM) significantly reduced detyrosinated tubulin (Detyr) levels compared to vehicle (*P <0.05; ** P <0.001, t‒test). Resveratrol, a non‒sesquiterpene lactone NF‒κB inhibitor, did not significantly affect detyrosinated tubulin (P >0.5, t‒test). Taxol significantly increased detyrosinated tubulin (‡Bt‒549 Tax value is × 2.5; MDA‒MB‒157 Tax value is × 10). None of the compounds induced apoptosis in these cells, as gauged by PARP cleavage. Columns, mean densitometry for N = 3 (Bt‒549) or N = 6 (MDA‒MB‒157) experiments; bars, SD. **(B)** Comparison of the compounds used show parthenolide and costunolide, two sesquiterpene lactones, are structurally similar while the other compounds are structurally dissimilar. DMSO, dimethyl sulfoxide; NF-κB, nuclear factor-kappaB; PARP, poly(ADP-ribose) polymerase.

Human breast carcinoma cell lines, MDA-MB-157, Bt-549 and MDA-MB-436 were treated with anti-inflammatory or microtubule-targeting drugs to determine their effect on detyrosinated tubulin. These cell lines were selected based on their metastatic properties as well as our previous research demonstrating high McTN frequencies and detyrosination of α-tubulin [12]. Following six hours of treatment, all cell lines show a significant dose-dependent decrease in detyrosinated tubulin in the presence of 10 to 25 μM of Parth and Cost (Figure 1A and B) (Bt-549: Parth10 (*P* <0.001), Parth25 (*P* < .001); Cost5 (*P* <0.05), Cost10 (*P* <0.05), Cost25 (*P* <0.001)), (MDA-MB-157: Parth10 (*P* <0.001), Parth25 (*P* <0.001); Cost10 (*P* <0.001), Cost25 (*P* <0.05)). The absence of PARP cleavage indicates that this concentration range did not reduce detyrosinated tubulin as a result of apoptosis. Treatment with ResV, a non-sesquiterpene lactone NF-κB inhibitor (Figure 1A and B), did not show a significant decrease (MDA-MB-157 and Bt-549 = *P* >0.5) in detyrosinated tubulin suggesting that the effects of Parth and Cost on detyrosinated tubulin are independent of their NF-κB inhibitory properties. Traditional microtubule-targeting drugs were included to show the effect on detyrosinated tubulin when microtubules are either destabilized (Col) or stabilized (Tax). The six-hour timepoint was chosen based on prior reports that have shown that the NF-κB inhibitory effects of Parth, Cost and ResV are realized within a three- to eight-hour window [22,23] as well as a time course of Parth and Cost in the experimental cell lines to determine when detyrosinated tubulin was significantly reduced [see Additional file 1: Figure S1]. Comparison of the drug structures shows strong similarity between Parth and Cost, which differ only in an epoxide group, while the other compounds are structurally dissimilar (Figure 1B).

Previous research using a novel high-throughput cell-based immunoluminescence assay has shown that Parth, but not Cost, can prevent accumulation of detyrosinated tubulin purportedly through TCP inhibition following a two hour treatment duration; however, these experiments were conducted in HeLa cells which have undetectable levels of detyrosinated microtubules (13). We report here that both Parth and Cost can significantly reduce detyrosinated tubulin in multiple invasive breast carcinomas with abundant detyrosinated microtubules. Our observation that Cost can reduce detyrosinated tubulin similarly to Parth may indicate that breast cancer cell lines with increased detyrosination levels are more susceptible to inhibition, or that extending treatment beyond two hours is required to realize the inhibitory action of Cost [see Additional file 1: Figure S1].

### Tubulin detyrosination is independent of the NF-κB pathway

**Figure 2:**
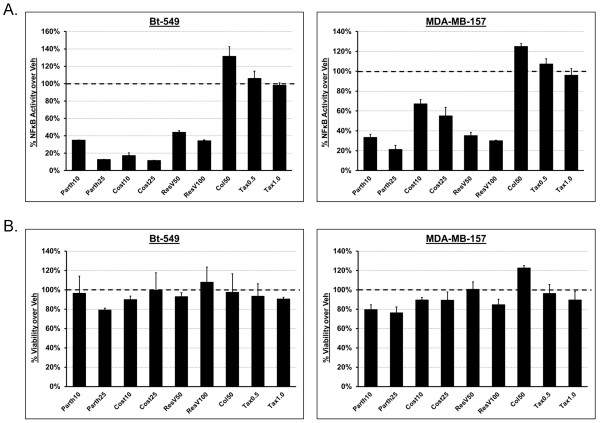
**Tubulin detyrosination is independent of NF-κB activation. (A)** NF-κB -luciferase reporter adenovirus infected MDA-MB-157 and Bt-549 show that a four-hour treatment of parthenolide (Parth; 10 μM, 25 μM) and costunolide (Cost; 10 μM, 25 μM) inhibit TNF-α-induced (100 ng/ml) NF-κB activation at concentrations that reduced detyrosinated tubulin. Resveratrol (ResV; 50 μg/ml, 100 μg/ml) inhibits NF-κB but does not affect detyrosination. Colchicine (Col; 50 μM) and Taxol (Tax; 0.5 μg/ml) do not inhibit NF-κB at concentrations that affect detyrosinated tubulin. **(B)** Cell viability assay shows that non-toxic drug concentrations are used. All compounds are expressed as a % of vehicle (set at 100%; horizontal dotted line). NF-κB, nuclear factor-kappaB.

Since normal cells are not generally as sensitive as tumor cells to sesquiterpene lactones due to low basal NF-κB activity, these compounds may target cancers in which the NF-κB pathway is constitutively activated. Studies have also shown that NF-κB activity has been associated with the polymerization state of microtubules. Therefore, we used an NF-κB reporter assay to determine if the effects of Parth and Cost on detyrosinated tubulin are related to their anti-inflammatory properties. Parth and Cost effectively inhibit TNF-α-induced NF-κB activation (Figure 2A, Additional file 2: Figure S2A) at the concentrations that reduce detyrosinated tubulin (Figure 1A). ResV also inhibited NF-κB (Figure 2A, Additional file 2: Figure S2A) but did not affect detyrosinated tubulin (Figure 1A), highlighting that the reduction in detyrosinated tubulin is independent of NF-κB inhibition. Tax increased detyrosinated tubulin but did not inhibit or synergistically increase TNF-α activation of NF-κB in all cell lines. Interestingly, Col decreased detyrosinated tubulin but caused a moderate increase in the NF-κB activation in all lines, suggesting that microtubule depolymerization may activate NF-κB as previously reported. Furthermore, the drug concentrations used did not affect viability (Figure 2B, Additional file 2: Figure S2B). These results indicate that the effects on detyrosinated tubulin are independent of NF-κB activation, revealing novel anticancer properties of these two sesquiterpene lactones.

### Parthenolide and costunolide decrease detyrosinated tubulin without compromising the overall microtubule network

**Figure 3:**
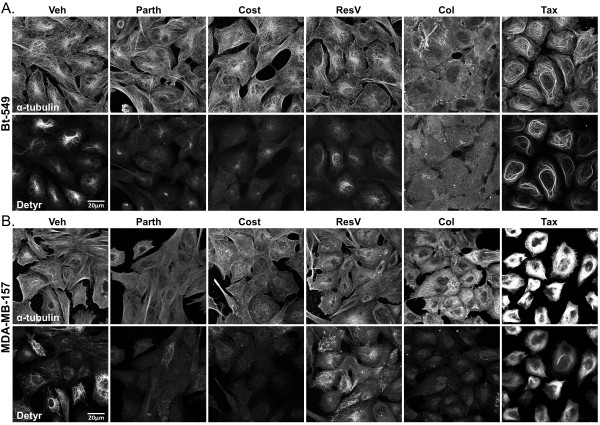
**Parthenolide and costunolide decrease detyrosinated tubulin without compromising the overall microtubule network.** Immunofluorescence of six-hour drug treated **(A)** Bt-549 and **(B)** MDA-MB-157 stained for detyrosinated tubulin and α-tubulin show that parthenolide (Parth; 10 μM) and costunolide (Cost; 25 μM) decrease and disrupt filamentous detyrosinated tubulin (Detyr) while leaving the overall microtubule network intact (α-tub). Treatment with colchicine (Col; 50 μM) disrupts both detyrosinated and α-tubulin filaments. Taxol (Tax; 0.5 μg/ml) increases bundling and disrupts organization of detyrosinated and α-tubulin filaments. Resveratrol (ResV; 50 μg/ml) did not significantly affect detyrosinated or α-tubulin.

While detyrosinated microtubules have been implicated in tumor aggressiveness, exclusive targeting of this post-translational modification with effective small molecule compounds has been a greater challenge. The majority of current anti-tubulin agents bind directly to tubulin and affect microtubule dynamics, ultimately impairing cellular functions to an extent that inhibits proliferation and triggers apoptosis. While this is the desired outcome for cancer cells, the side effects can lead to toxicity in non-target cells resulting in clinical limitations. Therefore, immunofluorescence was performed to investigate the structural impact of Parth and Cost on detyrosinated microtubules relative to the overall microtubule network. The vehicle treatment (0.1% DMSO) did not affect the elaborate filamentous detyrosinated tubulin that was observed in nearly every cell in the population (Figure 3A and B). ResV did not affect either detyrosinated tubulin or the microtubule network. The microtubule destabilizing drug Col significantly disrupted the detyrosinated microtubules while also destroying the overall microtubule array. Tax increased cellular levels and bundling, and altered the organization of detyrosinated microtubules while also affecting the microtubule network. Tax also caused cell shrinkage in both lines. Interestingly, Parth and Cost decreased detyrosinated microtubules without disrupting the overall microtubule network, demonstrating that these compounds have more targeted effects on stabilized microtubules than either Col or Tax. These results, in combination with the western blot, show that Parth and Cost selectively decrease detyrosinated microtubules.

### Parthenolide and costunolide decrease microtentacle (McTN) frequency

**Figure 4:**
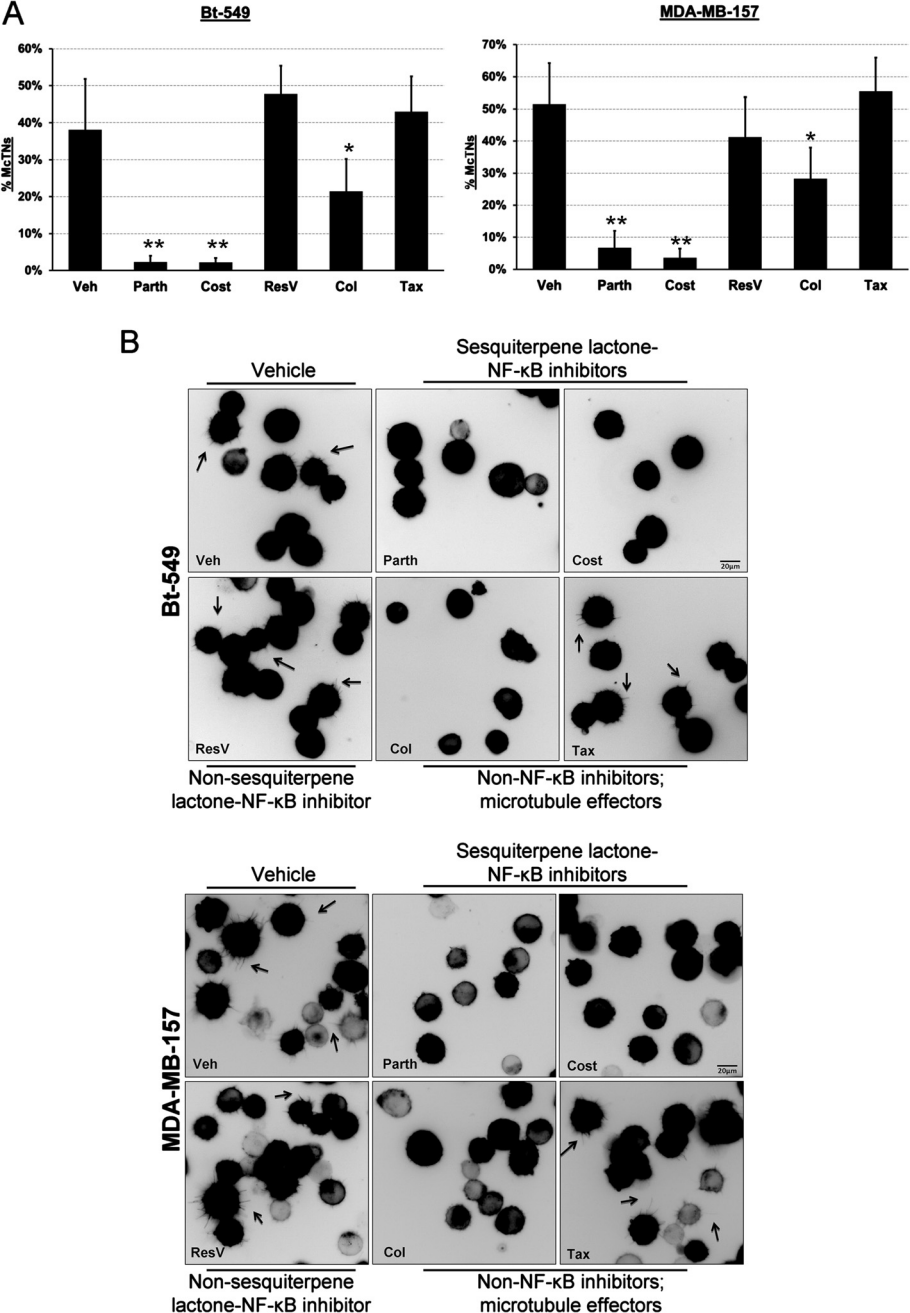
**Parthenolide and costunolide decrease microtentacle (McTN) frequency and attachment. (A)** Detached Bt-549 and MDA-MB-157 were pretreated for six hours and suspended in drug-containing media for blind McTN scoring. Parthenolide (Parth; 10 μM), costunolide (Cost; 25 μM), and colchicine (Col; 50 μM) show a significant decrease compared to vehicle treated (*P <0.05; ** *P* <0.001, t-test). Resveratrol (ResV; 50 μg/ml) and Taxol (Tax; 0.5 μg/ml) did not have a significant effect on McTN frequency. Columns, mean %McTNs for six independent experiments in which at least 100 cells were scored blindly; bars, SD. **(B)** Live population images of suspended Bt-549 and MDA-MB-157. McTNs are observed in vehicle, resveratrol, and Taxol (black arrows).

Our previous research has identified detyrosinated α-tubulin as a structural component of McTNs observed in detached cells. Additionally, increased McTN frequency has been correlated with invasiveness in a panel of breast tumor cell lines [12]. Based on Parth and Cost’s ability to selectively reduce detyrosinated tubulin, we investigated their effects on McTN frequency using a panel of invasive human breast carcinoma cells with high McTN frequency. Following six hours of treatment, cells infected with a membrane-targeted GFP were detached in drug-containing media and McTNs were scored blindly. Parth and Cost significantly (all lines: *P* <0.001) reduced McTN frequency to a greater extent than Col (Figure 4A; Additional file 3: Figure S3A). ResV and Tax did not change McTN frequency compared to the vehicle control (Figure 4A and B, Additional file 3: Figure S3A and B). Live-cell imaging of the detached population illustrates the considerable reduction in McTNs in the presence of Parth and Cost (Figure 4B, Additional file 3: Figure S3B).

### Parthenolide and costunolide reduce tumor cell attachment

**Figure 5:**
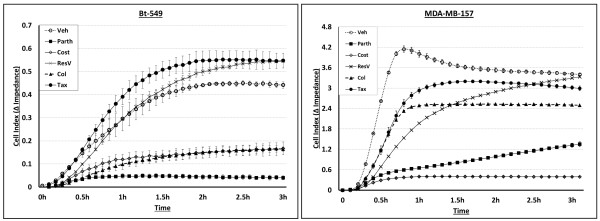
**Parthenolide and costunolide reduce reattachment efficiency of human breast carcinoma cells.** Real-time electrical impedance monitoring shows that parthenolide (10 μM) and costunolide (25 μM) significantly reduce attachment when compared to vehicle while Taxol (0.5 μg/ml) and resveratrol (50 μg/ml) did not. Colchicine (50 μM) reduced attachment to a greater extent in Bt-549 than in MDA-MB-157. Lines, mean for three triplicate wells; bars, SD; representative graph is shown. Three independent experiments were performed [see Additional file 4: Figure S4].

We previously showed that McTNs facilitate cell attachment to ECM and to an endothelial layer [11]. Additionally, mesenchymal cells with increased detyrosinated tubulin and McTNs attach at a faster rate than their epithelial counterpart [11]. Given the significant reductions in McTN frequencies and selective targeting of detyrosinated tubulin by Parth and Cost, we compared the reattachment efficiency of each line in the presence of drug using real-time electrical impedance monitoring. Each cell line was pretreated for six hours and then plated into media containing the respective drug in specialized E-plates with sensor electrode arrays. Real-time attachment monitoring shows that Parth and Cost inhibited cells from reattaching in all lines while ResV and Tax attached similarly to the vehicle control (Figure 5; Additional file 4: Figure S4 and Additional file 5: Figure S5). Interestingly, Col treatment decreased attachment in Bt-549 and MDA-MB-436 more strongly than in MDA-MB-157, illustrating differences in cell sensitivity. These results suggest that Parth and Cost reduced attachment by affecting detyrosinated tubulin, a major structural component of McTNs.

## Discussion

Metastasis causes 90% of deaths from solid tumors; therefore, novel chemotherapeutic strategies that go beyond primary tumor treatment and inhibit metastatic dissemination are critical. Since cancer cells can be shed from primary tumors that remain below the threshold of detection [24] and microtubules can influence the metastatic cascade, the effects of microtubule-directed compounds on circulating tumor cells are a crucial area for investigation. Interestingly, treatment with a tubulin depolymerizing agent prevents circulating colon carcinoma cells from attaching to the microvascular endothelium *in vivo,* highlighting a microtubule-dependent mechanism for circulating tumor cell retention in distant tissues [25]. Recent patient studies have further shown a rapid increase of circulating tumor cells in the bloodstream with neoadjuvant taxane treatment and a two-fold higher frequency of relapse compared to adjuvant taxane treatment [24]. Furthermore, higher paclitaxel concentrations have been correlated with increased survival in some tumor cells compared to lower doses [26]. Research from our laboratory has identified tubulin-based McTNs in detached cells that facilitate reattachment to endothelial layers, a tubulin-driven mechanism that possibly connects the data observed *in vivo* and in patients treated with microtubule stabilizers. These data necessitate a strategic and cautious approach for selecting the most appropriate patient treatment when circulating tumor cells are present.

Our present results show that Parth and Cost can reduce McTNs by specifically targeting detyrosinated tubulin unlike traditional tubulin-targeted compounds, Tax and Col. These observations support previous evidence showing that Tax alone is insufficient to stimulate the formation of new McTNs in invasive cell lines but can stabilize existing structures to promote MT-dependent adhesion and cell spreading [27]. Interestingly, conditions where filamentous actin is disrupted within the cell show a robust increase in stable microtubules [28] and McTN frequency when treated with Tax [27], highlighting the consequences when the microtubule-microfilament interaction is unbalanced and stable microtubules are unrestricted. This evidence is of particular relevance given data showing that EMT destabilizes cortical actin [29] and that malignant cells have a 40% reduction in filamentous actin [30]. Furthermore, microtubules can continue to grow after membrane contact, divert along the plasma membrane, push the membrane outward, and even grow inward in TTL knockout cells [10], conditions that increase detyrosinated tubulin and promote invasiveness. Therefore, the effects of indiscriminate disruption of major cytoskeletal networks may contribute to metastasis or toxic side effects.

Chronic tissue damage and inflammation have been associated with tumor development as well as EMT [13,31,32], conditions that may promote persistent microtubule stability and aberrant detyrosinated tubulin elevation. Persistent stimuli that activate inflammatory pathways and elicit a microtubule stabilization response may provide selective pressure for mutations that suppress TTL activity and/or upregulate the TCP. Interestingly, suppression of TTL has been associated with poor prognosis in several cancers [7-9]. Due to the limited characterization of the TCP or resources to measure its expression, it is unclear what alterations in the TCP might exist in cancer cells to increase microtubule stability and possibly provide the cell a selective advantage. Only recently has AGLB2 been identified as the TCP that regulates the tubulin tyrosination cycle by interacting with retinoic acid receptor responder 1 (RARRES1), a carboxypeptidase inhibitor that is suppressed in aggressive prostate and breast cancer cells with a mesenchymal phenotype [33]. Nevertheless, research has shown that detyrosinated microtubules are oriented towards a wound site [4] in addition to being upregulated at the tumor invasive front [11]. This increase in tubulin detyrosination supports cell migration, proliferation and EMT, likely influencing metastatic success and chemoresistance. Once detached and disseminated from the primary tumor, a circulating tumor cell can reattach at a distant site, a process proposed to be microtubule-driven [25,34]. Therefore, compounds that can reduce detyrosinated tubulin as well as inflammation could be a multi-pronged approach for cancer treatment as well as prevention.

There is growing evidence that select nonsteroidal anti-inflammatory drugs (NSAID) have anti-cancer properties, although the mechanisms are still being elucidated [35]. We show that the NSAIDs Parth, Cost and ResV can inhibit TNF-α activation of NF-κB; however, only Parth and Cost can selectively reduce detyrosinated tubulin highlighting that NF-κB inhibition is independent of effects on tubulin detyrosination. Parth and Cost could be capable of modulating the enzymatic reaction responsible for tubulin detyrosination [18] and reduce McTNs without total microtubule disruption. Modulating microtubule stability with alternative chemotherapeutics may also enable the benefits of Tax treatment to be realized with combination therapy [36]. Supporting data indicate that Parth has displayed a significantly better prognosis in combination therapy with paclitaxel than paclitaxel alone *in vivo* using human gastric cancer cells as well as breast cancer cells [37,38]. The combination treatment with Parth *in vitro* using non-small cell lung cancer lowered the effective Tax dose required to induce cytotoxicity [39] as well as resensitized Tax-resistant cells [40]. Moreover, NSAID sesquiterpene lactones in cancer clinical trials, including Parth, have displayed selectivity to target tumor and cancer stem cells while sparing normal cells [38]. Of particular relevance is Parth’s ability to suppress the formation of disseminated nodules *in vivo* (gastric cancer) and inhibit bone metastasis of W256 breast cancer cells in mice [41]. Cost has also shown antiproliferative activity in leukemia cells as well as efficacy in drug resistant lines [42] but has been studied to a lesser extent than Parth. Given detyrosinated tubulin’s role in cell migration and reattachment, it is possible that Parth’s ability to reduce detyrosinated tubulin and McTN formation is a contributing factor to the inhibition of metastasis, a mechanism that is independent from its anti-inflammatory effects.

Most clinical microtubule-targeted drugs bind directly to tubulin, resulting in an increase or decrease in the microtubule mass [43]. It has been noted that tubulin binding and subsequent dynamic suppression contribute to the benefits but also to the toxic side effects of these drugs [2]. Intact microtubule arrays are necessary for normal cell functions, so it is expected that disrupting the microtubule network will have significant effects on both cancer and normal cells. For example, it has been reported that Col has such high toxicity to normal tissues that its development as an anticancer therapeutic has been unsuccessful [2]. Encouragingly, it has been previously shown that it is possible to interfere with microtubule dynamics without dismantling the entire microtubule array in order to successfully reduce migration [44]. Our data show that both Col and Tax severely affect the microtubule network while Parth and Cost selectively reduce a subset of detyrosinated, stable microtubules that are associated with tumor aggressiveness and EMT. The therapeutic goal is to reduce the broad toxicity associated with many of the microtubule chemotherapeutics and direct the treatment at malignant cell hallmarks. Therefore, the enzymes regulating the detyrosination/tyrosination cycle could be an appealing and more specific drug target to moderate microtubule stability in tumors with high levels of detyrosinated tubulin rather than broadly targeting tubulin polymerization with compounds that bind tubulin subunits directly.

## Conclusions

Based on the developing evidence, we propose that circulating tumor cell reattachment is facilitated by detyrosinated tubulin enriched McTNs [25,34]. Detyrosinated tubulin may favor aberrant microtubule stability associated with cell migration [4], proliferation [45], chemoresistance [4], EMT [11], McTN formation [12], reattachment [11,25] and tumor aggressiveness [7]. Our data demonstrate that targeting detyrosinated tubulin with compounds such as Parth and Cost can reduce McTN frequency and inhibit reattachment without significantly disrupting the overall microtubule network or cell viability. These actions are independent of their effects on NF-κB inhibition presenting a novel property to these sesquiterpene lactones and an opportunity to move beyond simply targeting tumor cell growth. Our results highlight Parth and Cost as potential compounds to target detyrosinated tubulin more selectively. Since this α-tubulin modification promotes McTNs and tumor cell reattachment, such treatment could reduce tumor aggressiveness and decrease the risk of recurrence with less toxicity in breast cancer patients.

## Abbreviations

Col: colchicine; Cost: costunolide; EMT: epithelial-to-mesenchymal transition; GFP: green fluorescent protein; McTN: microtentacle; NF-κB: nuclear factor-kappaB; PARP: poly(ADP-ribose) polymerase; Parth: parthenolide; ResV: resveratrol; Tax: paclitaxel; TCP: tubulin carboxypeptidase; TNF-α: tumor necrosis factor alpha; TTL: tubulin tyrosine ligase.

## Competing interests

The authors declare that they have no competing interests.

## Authors’ contributions

RW and MV designed the experiments and analyzed the data. RW carried out the experiments. AB assisted with the live cell imaging and immunofluorescence. MC also assisted in the experimental design. KT performed some of the preliminary research experiments. RW, MV, AB, MC, KT and SM participated in drafting and editing the manuscript. SM conceived the study and participated in the experimental design. All authors have read and approved the final manuscript.

## Supplementary Material

Additional file 1: Figure S1 Bt-549 and MDA-MB-157 cells treated for one hour, three hours and six hours with parthenolide (Parth; 10 μM), and costunolide (Cost; 25 μM) show that detyrosinated tubulin is significantly reduced by six hours compared to DMSO (Veh; 0.1%) treated cells (N = 3). Click here for file

Additional file 2: Figure S2 (A) NF-κB -luciferase reporter adenovirus infected MDA-MB-436 shows that a four hour treatment of parthenolide (Parth; 10 μM, 25 μM) and costunolide (Cost; 10 μM, 25 μM) inhibits TNF-α-induced (100 ng/ml) NF-κB activation at concentrations that reduced detyrosinated tubulin. Resveratrol (ResV; 50 μg/ml, 100 μg/ml) inhibits NF-κB but does not affect detyrosination. Colchicine (Col; 50 μM) and Taxol (Tax; 0.5 μg/ml) do not inhibit NF-κB at concentrations that affect detyrosinated tubulin. (B) Cell viability assay shows that non-toxic drug concentrations are used. All compounds are expressed as a % of vehicle (set at 100%; horizontal dotted line). Click here for file

Additional file 3: Figure S3 (A) Detached MDA-MB-436 were pretreated for six hours and suspended in drug containing media for blind McTN scoring. Parthenolide (Parth; 10 μM), costunolide (*Cost*; 25 μM), and colchicine (Col; 50 μM) show a significant decrease compared to vehicle treated (*P <0.05; ** *P* <0.001, *t*-test). Resveratrol (ResV; 50 μg/ml) and Taxol (Tax; 0.5 μg/ml) did not have a significant effect on McTN frequency. Columns, mean %McTNs for four independent experiments in which at least 100 cells were scored blindly; bars, SD. (B) Live population images of suspended MDA-MB-436. McTNs are observed in vehicle, resveratrol, and Taxol (black arrows). Click here for file

Additional file 4: Figure S4 Real-time electrical impedance monitoring of MDA-MB-436 shows that parthenolide (10 μM) and costunolide (25 μM) significantly reduce attachment when compared to vehicle while Taxol (0.5 μg/ml) and resveratrol (50 μg/ml) did not. Colchicine (50 μM) also reduced attachment. Lines, mean for three triplicate wells; bars, SD; representative graph is shown. Click here for file

Additional file 5: Figure S5 Additional real-time electrical impedance monitoring experiment trials of Bt-549 and MDA-MB-157 showing that parthenolide (10 μM) and costunolide (25 μM) significantly reduce attachment when compared to vehicle while Taxol (0.5 μg/ml) and resveratrol (50 μg/ml) did not. Colchicine (50 μM) reduced attachment to a greater extent in Bt-549 than in MDA-MB-157. Lines, mean for three triplicate wells; bars, SD. Click here for file

## References

[B1] de Forges H Bouissou A Perez F Interplay between microtubule dynamics and intracellular organization Int J Biochem Cell Biol 2012 15 266 274 10.1016/j.biocel.2011.11.009 22108200

[B2] Risinger AL Giles FJ Mooberry SL Microtubule dynamics as a target in oncology Cancer Treat Rev 2009 15 255 261 10.1016/j.ctrv.2008.11.001 19117686 PMC2778221

[B3] Singh P Rathinasamy K Mohan R Panda D Microtubule assembly dynamics: an attractive target for anticancer drugs IUBMB Life 2008 15 368 375 10.1002/iub.42 18384115

[B4] Gundersen GG Bulinski JC Selective stabilization of microtubules oriented toward the direction of cell migration Proc Natl Acad Sci U S A 1988 15 5946 5950 10.1073/pnas.85.16.5946 3413068 PMC281882

[B5] Kirschner M Mitchison T Beyond self-assembly: from microtubules to morphogenesis Cell 1986 15 329 342 10.1016/0092-8674(86)90318-1 3516413

[B6] Gundersen GG Khawaja S Bulinski JC Generation of a stable, posttranslationally modified microtubule array is an early event in myogenic differentiation J Cell Biol 1989 15 2275 2288 10.1083/jcb.109.5.2275 2681230 PMC2115884

[B7] Mialhe A Lafanechere L Treilleux I Peloux N Dumontet C Bremond A Panh MH Payan R Wehland J Margolis RL Job D Tubulin detyrosination is a frequent occurrence in breast cancers of poor prognosis Cancer Res 2001 15 5024 5027 11431336

[B8] Lafanechere L Courtay-Cahen C Kawakami T Jacrot M Rudiger M Wehland J Job D Margolis RL Suppression of tubulin tyrosine ligase during tumor growth J Cell Sci 1998 15 171 181 9405300 10.1242/jcs.111.2.171

[B9] Soucek K Kamaid A Phung AD Kubala L Bulinski JC Harper RW Eiserich JP Normal and prostate cancer cells display distinct molecular profiles of alpha-tubulin posttranslational modifications Prostate 2006 15 954 965 10.1002/pros.20416 16541425

[B10] Peris L Wagenbach M Lafanechere L Brocard J Moore AT Kozielski F Job D Wordeman L Andrieux A Motor-dependent microtubule disassembly driven by tubulin tyrosination J Cell Biol 2009 15 1159 1166 10.1083/jcb.200902142 19564401 PMC2712961

[B11] Whipple RA Matrone MA Cho EH Balzer EM Vitolo MI Yoon JR Ioffe OB Tuttle KC Yang J Martin SS Epithelial-to-mesenchymal transition promotes tubulin detyrosination and microtentacles that enhance endothelial engagement Cancer Res 2010 15 8127 8137 10.1158/0008-5472.CAN-09-4613 20924103 PMC3123454

[B12] Whipple RA Balzer EM Cho EH Matrone MA Yoon JR Martin SS Vimentin filaments support extension of tubulin-based microtentacles in detached breast tumor cells Cancer Res 2008 15 5678 5688 10.1158/0008-5472.CAN-07-6589 18632620 PMC2859318

[B13] Lopez-Novoa JM Nieto MA Inflammation and EMT: an alliance towards organ fibrosis and cancer progression EMBO Mol Med 2009 15 303 314 10.1002/emmm.200900043 20049734 PMC3378143

[B14] Chua HL Bhat-Nakshatri P Clare SE Morimiya A Badve S Nakshatri H NF-kappaB represses E-cadherin expression and enhances epithelial to mesenchymal transition of mammary epithelial cells: potential involvement of ZEB-1 and ZEB-2 Oncogene 2007 15 711 724 10.1038/sj.onc.1209808 16862183

[B15] Julien S Puig I Caretti E Bonaventure J Nelles L van Roy F Dargemont C de Herreros AG Bellacosa A Larue L Activation of NF-kappaB by Akt upregulates Snail expression and induces epithelium mesenchyme transition Oncogene 2007 15 7445 7456 10.1038/sj.onc.1210546 17563753

[B16] Bocca C Gabriel L Bozzo F Miglietta A A sesquiterpene lactone, costunolide, interacts with microtubule protein and inhibits the growth of MCF-7 cells Chem Biol Interact 2004 15 79 86 10.1016/j.cbi.2003.10.008 14726154

[B17] Miglietta A Bozzo F Gabriel L Bocca C Microtubule-interfering activity of parthenolide Chem Biol Interact 2004 15 165 173 10.1016/j.cbi.2004.07.005 15501437

[B18] Fonrose X Ausseil F Soleilhac E Masson V David B Pouny I Cintrat JC Rousseau B Barette C Massiot G Lafanechère L Parthenolide inhibits tubulin carboxypeptidase activity Cancer Res 2007 15 3371 3378 10.1158/0008-5472.CAN-06-3732 17409447

[B19] Mackenzie GG Keen CL Oteiza PI Microtubules are required for NF-kappaB nuclear translocation in neuroblastoma IMR-32 cells: modulation by zinc J Neurochem 2006 15 402 415 10.1111/j.1471-4159.2006.04005.x 17029595

[B20] Rosette C Karin M Cytoskeletal control of gene expression: depolymerization of microtubules activates NF-kappa B J Cell Biol 1995 15 1111 1119 10.1083/jcb.128.6.1111 7896875 PMC2120413

[B21] Aggarwal BB Shishodia S Takada Y Banerjee S Newman RA Bueso-Ramos CE Price JE Curcumin suppresses the paclitaxel-induced nuclear factor-kappaB pathway in breast cancer cells and inhibits lung metastasis of human breast cancer in nude mice Clin Cancer Res 2005 15 7490 7498 10.1158/1078-0432.CCR-05-1192 16243823

[B22] Nakshatri H Rice SE Bhat-Nakshatri P Antitumor agent parthenolide reverses resistance of breast cancer cells to tumor necrosis factor-related apoptosis-inducing ligand through sustained activation of c-Jun N-terminal kinase Oncogene 2004 15 7330 7344 10.1038/sj.onc.1207995 15286701

[B23] Takada Y Bhardwaj A Potdar P Aggarwal BB Nonsteroidal anti-inflammatory agents differ in their ability to suppress NF-kappaB activation, inhibition of expression of cyclooxygenase-2 and cyclin D1, and abrogation of tumor cell proliferation Oncogene 2004 15 9247 9258 15489888 10.1038/sj.onc.1208169

[B24] Hekimian K Meisezahl S Trompelt K Rabenstein C Pachmann K Epithelial cell dissemination and readhesion: analysis of factors contributing to metastasis formation in breast cancer ISRN Oncol 2012 15 601810 doi: 10.5402/2012/601810 22530147 10.5402/2012/601810 PMC3317055

[B25] Korb T Schluter K Enns A Spiegel HU Senninger N Nicolson GL Haier J Integrity of actin fibers and microtubules influences metastatic tumor cell adhesion Exp Cell Res 2004 15 236 247 10.1016/j.yexcr.2004.06.001 15302590

[B26] Liebmann JE Cook JA Lipschultz C Teague D Fisher J Mitchell JB Cytotoxic studies of paclitaxel (Taxol) in human tumour cell lines Br J Cancer 1993 15 1104 1109 10.1038/bjc.1993.488 7903152 PMC1968657

[B27] Balzer EM Whipple RA Cho EH Matrone MA Martin SS Antimitotic chemotherapeutics promote adhesive responses in detached and circulating tumor cells Breast Cancer Res Treat 2010 15 65 78 10.1007/s10549-009-0457-3 19593636 PMC3633461

[B28] Bartolini F Ramalingam N Gundersen GG Actin-capping protein promotes microtubule stability by antagonizing the actin activity of mDia1 Mole Biol Cell 2012 15 4032 4040 10.1091/mbc.E12-05-0338 PMC3469518 22918941

[B29] Yilmaz M Christofori G EMT, the cytoskeleton, and cancer cell invasion Cancer Metastasis Rev 2009 15 15 33 10.1007/s10555-008-9169-0 19169796

[B30] Guck J Schinkinger S Lincoln B Wottawah F Ebert S Romeyke M Lenz D Erickson HM Ananthakrishnan R Mitchell D Käs J Ulvick S Bilby C Optical deformability as an inherent cell marker for testing malignant transformation and metastatic competence Biophys J 2005 15 3689 3698 10.1529/biophysj.104.045476 15722433 PMC1305515

[B31] Arwert EN Hoste E Watt FM Epithelial stem cells, wound healing and cancer Nature Rev Cancer 2012 15 170 180 10.1038/nrc3217 22362215

[B32] Mantovani A Allavena P Sica A Balkwill F Cancer-related inflammation Nature 2008 15 436 444 10.1038/nature07205 18650914

[B33] Sahab ZJ Hall MD Me Sung Y Dakshanamurthy S Ji Y Kumar D Byers SW Tumor suppressor RARRES1 interacts with cytoplasmic carboxypeptidase AGBL2 to regulate the alpha-tubulin tyrosination cycle Cancer Res 2011 15 1219 1228 10.1158/0008-5472.CAN-10-2294 21303978 PMC3062087

[B34] Whipple RA Cheung AM Martin SS Detyrosinated microtubule protrusions in suspended mammary epithelial cells promote reattachment Exp Cell Res 2007 15 1326 1336 10.1016/j.yexcr.2007.02.001 17359970 PMC3132414

[B35] Thun MJ Henley SJ Patrono C Nonsteroidal anti-inflammatory drugs as anticancer agents: mechanistic, pharmacologic, and clinical issues J Nat Cancer Inst 2002 15 252 266 10.1093/jnci/94.4.252 11854387

[B36] Ahmed AA Wang X Lu Z Goldsmith J Le XF Grandjean G Bartholomeusz G Broom B Bast RC Jr Modulating microtubule stability enhances the cytotoxic response of cancer cells to Paclitaxel Cancer Res 2011 15 5806 5817 10.1158/0008-5472.CAN-11-0025 21775522 PMC3679477

[B37] Sohma I Fujiwara Y Sugita Y Yoshioka A Shirakawa M Moon JH Takiguchi S Miyata H Yamasaki M Mori M Doki Y Parthenolide, an NF-kappaB inhibitor, suppresses tumor growth and enhances response to chemotherapy in gastric cancer Cancer Genomics Proteomics 2011 15 39 47 21289336

[B38] Zhou J Zhang H Gu P Bai J Margolick JB Zhang Y NF-kappaB pathway inhibitors preferentially inhibit breast cancer stem-like cells Breast Cancer Res Treat 2008 15 419 427 10.1007/s10549-007-9798-y 17965935 PMC3320112

[B39] Gao ZW Zhang DL Guo CB Paclitaxel efficacy is increased by parthenolide via nuclear factor-kappaB pathways in *in vitro* and *in vivo* human non-small cell lung cancer models Curr Cancer Drug Targets 2010 15 705 715 10.2174/156800910793605776 20578985

[B40] Gill KK Kaddoumi A Nazzal S Mixed micelles of PEG(2000)-DSPE and vitamin-E TPGS for concurrent delivery of paclitaxel and parthenolide: enhanced chemosenstization and antitumor efficacy against non-small cell lung cancer (NSCLC) cell lines Eur J Pharm Sci 2012 15 64 71 10.1016/j.ejps.2012.02.010 22369858

[B41] Idris AI Libouban H Nyangoga H Landao-Bassonga E Chappard D Ralston SH Pharmacologic inhibitors of IkappaB kinase suppress growth and migration of mammary carcinosarcoma cells *in vitro* and prevent osteolytic bone metastasis *in vivo* Mol Cancer Ther 2009 15 2339 2347 19671767 10.1158/1535-7163.MCT-09-0133

[B42] Choi JH Seo BR Seo SH Lee KT Park JH Park HJ Choi JW Itoh Y Miyamoto K Costunolide induces differentiation of human leukemia HL-60 cells Arch Pharm Res 2002 15 480 484 10.1007/BF02976606 12214860

[B43] Jordan MA Wilson L Microtubules as a target for anticancer drugs Nat Rev Cancer 2004 15 253 265 10.1038/nrc1317 15057285

[B44] Liao G Nagasaki T Gundersen GG Low concentrations of nocodazole interfere with fibroblast locomotion without significantly affecting microtubule level: implications for the role of dynamic microtubules in cell locomotion J Cell Sci 1995 15 3473 3483 8586659 10.1242/jcs.108.11.3473

[B45] Phung AD Soucek K Kubala L Harper RW Chloe Bulinski J Eiserich JP Posttranslational nitrotyrosination of alpha-tubulin induces cell cycle arrest and inhibits proliferation of vascular smooth muscle cells Eur J Cell Biol 2006 15 1241 1252 10.1016/j.ejcb.2006.05.016 17118269

