# Supplementary figures and images for: Parthenolide and costunolide reduce microtentacles and tumor cell attachment by selectively targeting detyrosinated tubulin independent from NF-κB inhibition
Source: Breast Cancer Res. 2013 Sep 13;15(5):R83. doi: 10.1186/bcr3477 (PMC3979133; doi:10.1186/bcr3477)

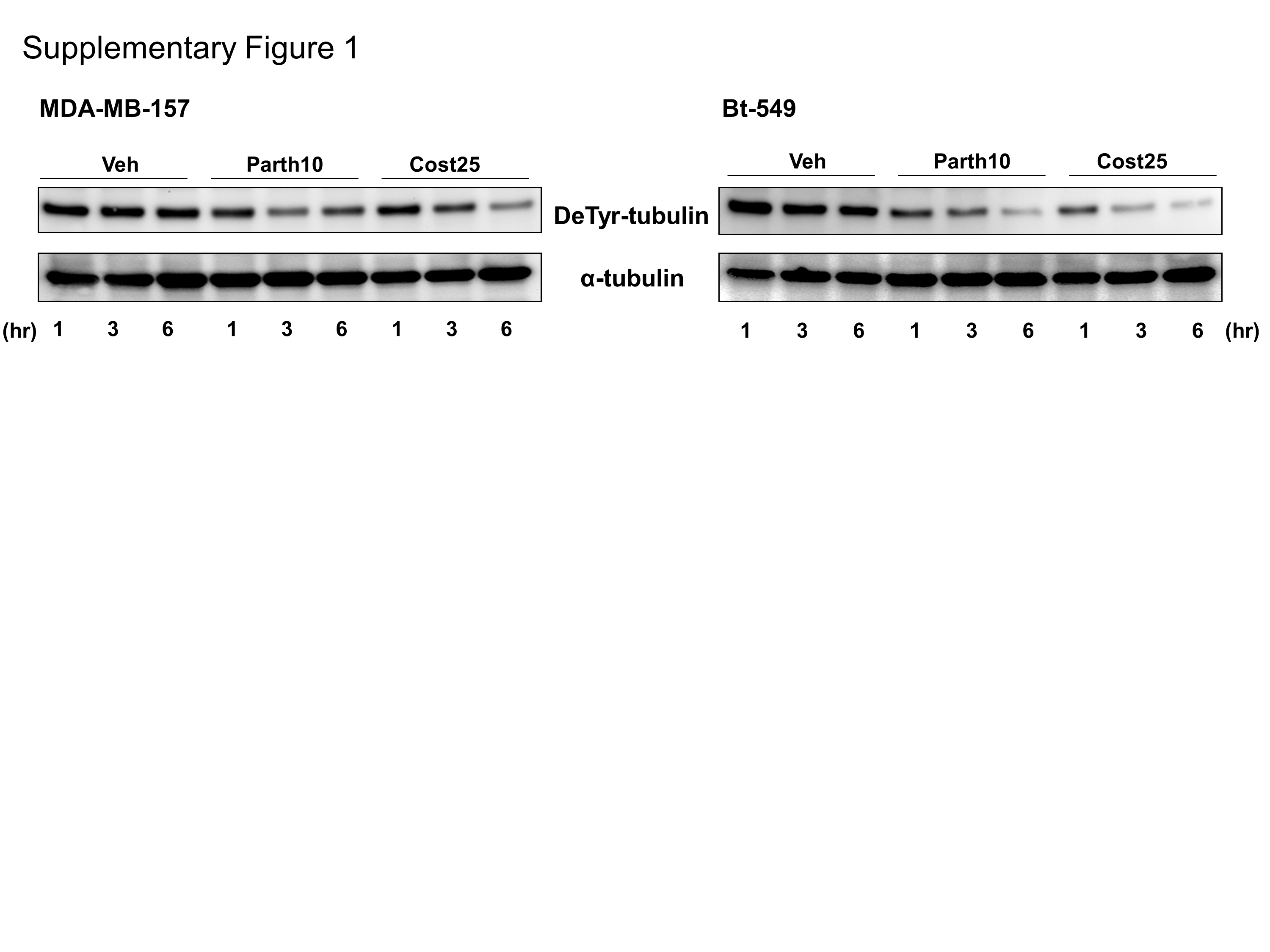

Supplement: Additional file 1: Figure S1 — Bt-549 and MDA-MB-157 cells treated for one hour, three hours and six hours with parthenolide (Parth; 10 μM), and costunolide (Cost; 25 μM) show that detyrosinated tubulin is significantly reduced by six hours compared to DMSO (Veh; 0.1%) treated cells (N = 3). [file bcr3477-S1.tiff]

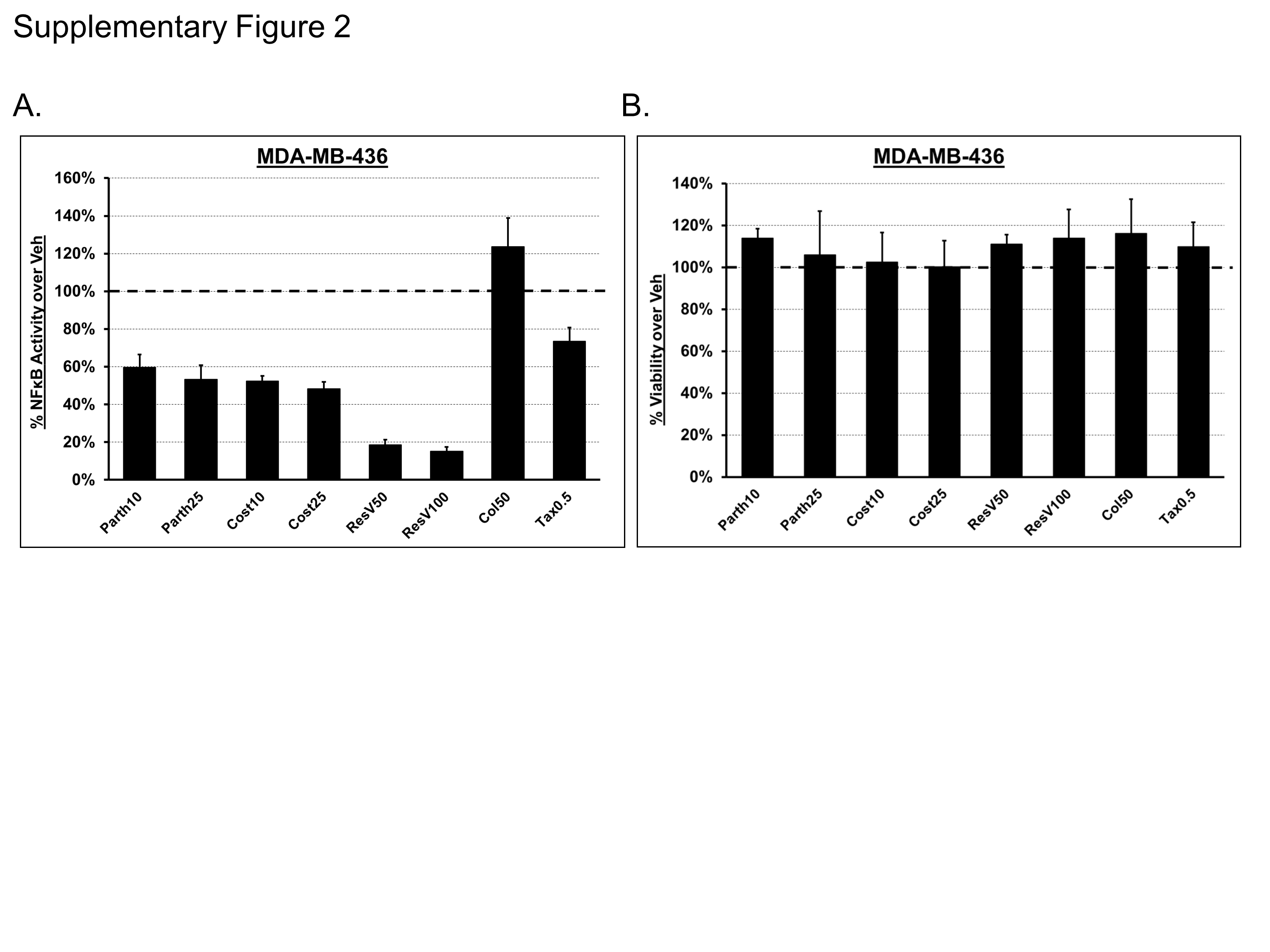

Supplement: Additional file 2: Figure S2 — (A) NF-κB -luciferase reporter adenovirus infected MDA-MB-436 shows that a four hour treatment of parthenolide (Parth; 10 μM, 25 μM) and costunolide (Cost; 10 μM, 25 μM) inhibits TNF-α-induced (100 ng/ml) NF-κB activation at concentrations that reduced detyrosinated tubulin. Resveratrol (ResV; 50 μg/ml, 100 μg/ml) inhibits NF-κB but does not affect detyrosination. Colchicine (Col; 50 μM) and Taxol (Tax; 0.5 μg/ml) do not inhibit NF-κB at concentrations that affect detyrosinated tubulin. (B) Cell viability assay shows that non-toxic drug concentrations are used. All compounds are expressed as a % of vehicle (set at 100%; horizontal dotted line). [file bcr3477-S2.tiff]

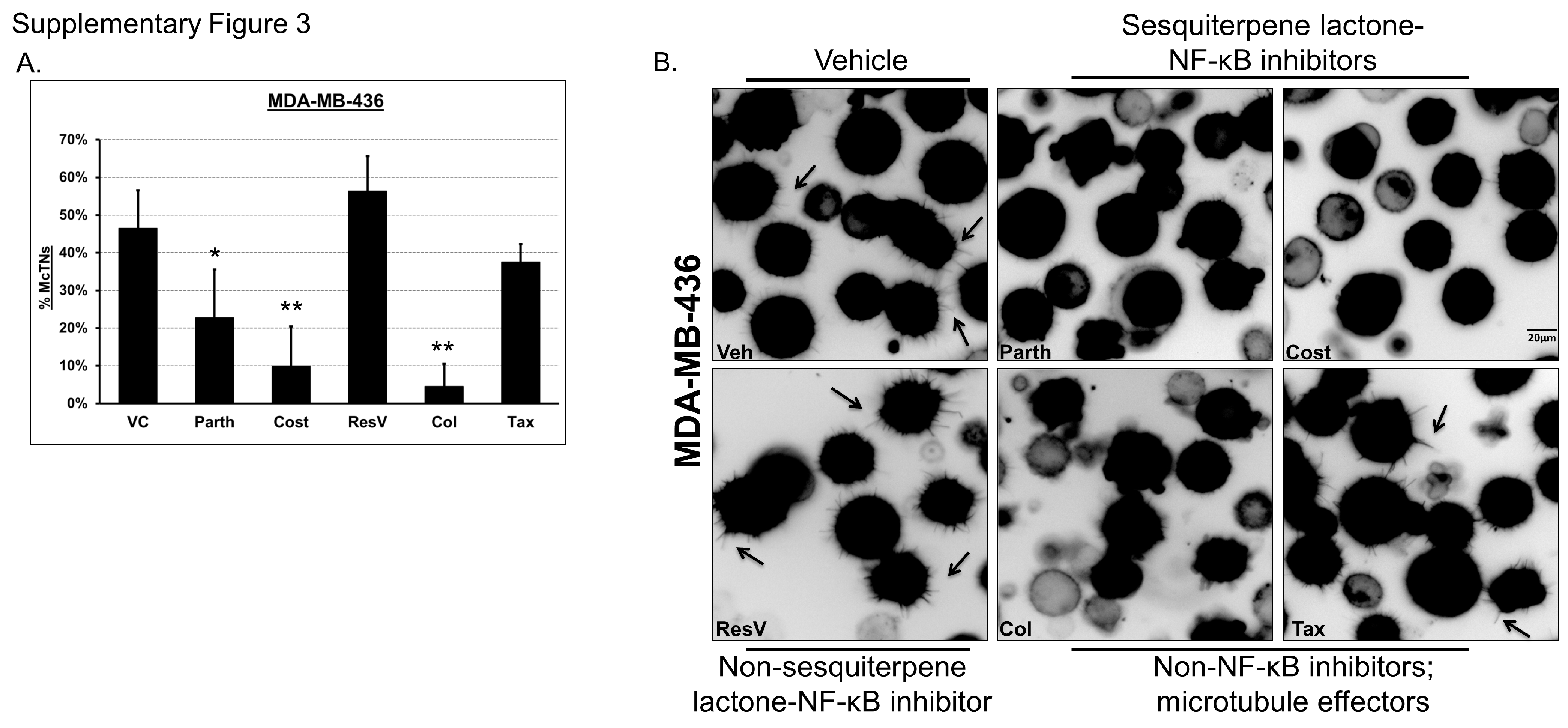

Supplement: Additional file 3: Figure S3 — (A) Detached MDA-MB-436 were pretreated for six hours and suspended in drug containing media for blind McTN scoring. Parthenolide (Parth; 10 μM), costunolide (Cost; 25 μM), and colchicine (Col; 50 μM) show a significant decrease compared to vehicle treated (*P <0.05; ** P <0.001, t-test). Resveratrol (ResV; 50 μg/ml) and Taxol (Tax; 0.5 μg/ml) did not have a significant effect on McTN frequency. Columns, mean %McTNs for four independent experiments in which at least 100 cells were scored blindly; bars, SD. (B) Live population images of suspended MDA-MB-436. McTNs are observed in vehicle, resveratrol, and Taxol (black arrows). [file bcr3477-S3.tiff]

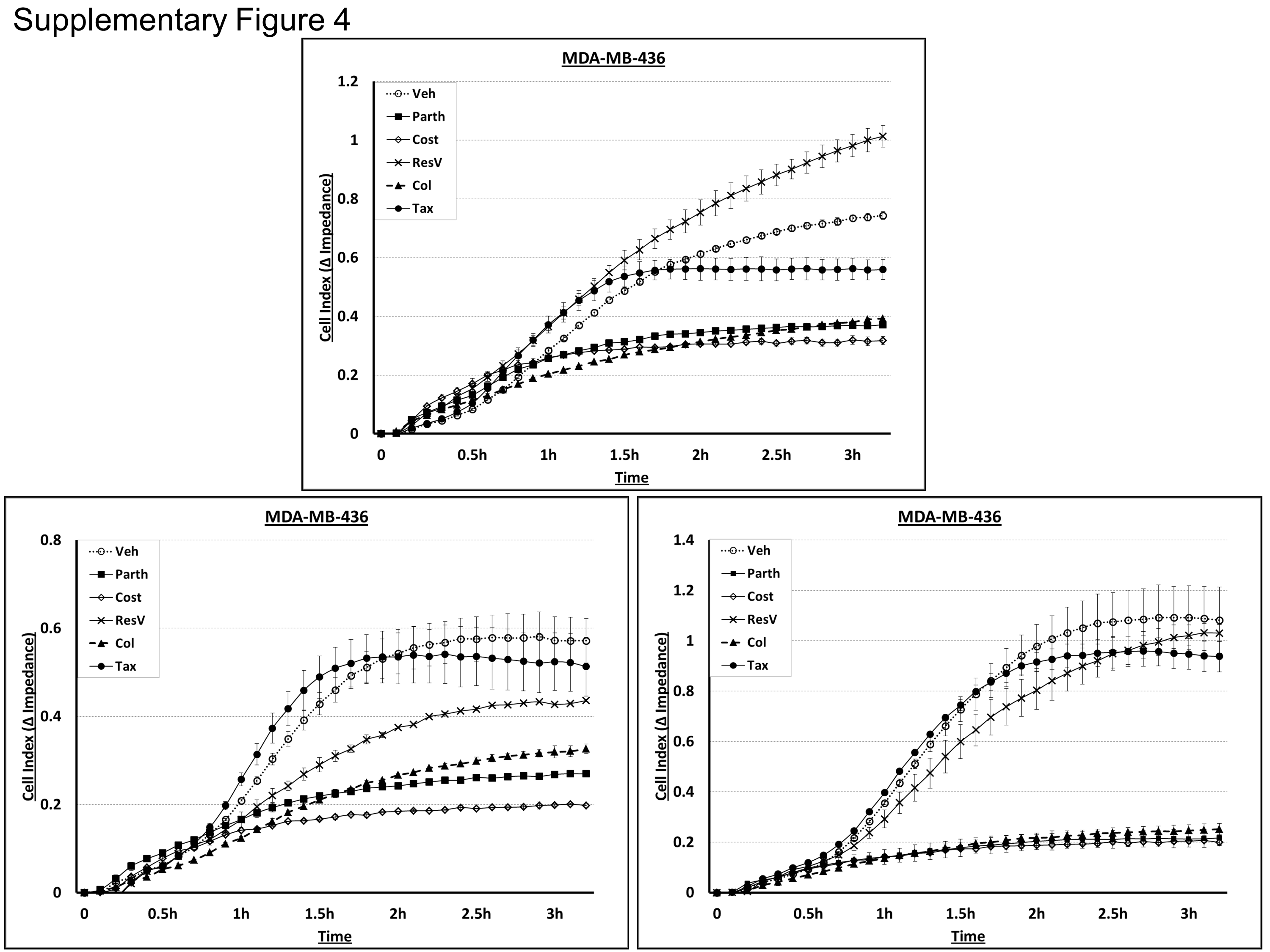

Supplement: Additional file 4: Figure S4 — Real-time electrical impedance monitoring of MDA-MB-436 shows that parthenolide (10 μM) and costunolide (25 μM) significantly reduce attachment when compared to vehicle while Taxol (0.5 μg/ml) and resveratrol (50 μg/ml) did not. Colchicine (50 μM) also reduced attachment. Lines, mean for three triplicate wells; bars, SD; representative graph is shown. [file bcr3477-S4.tiff]

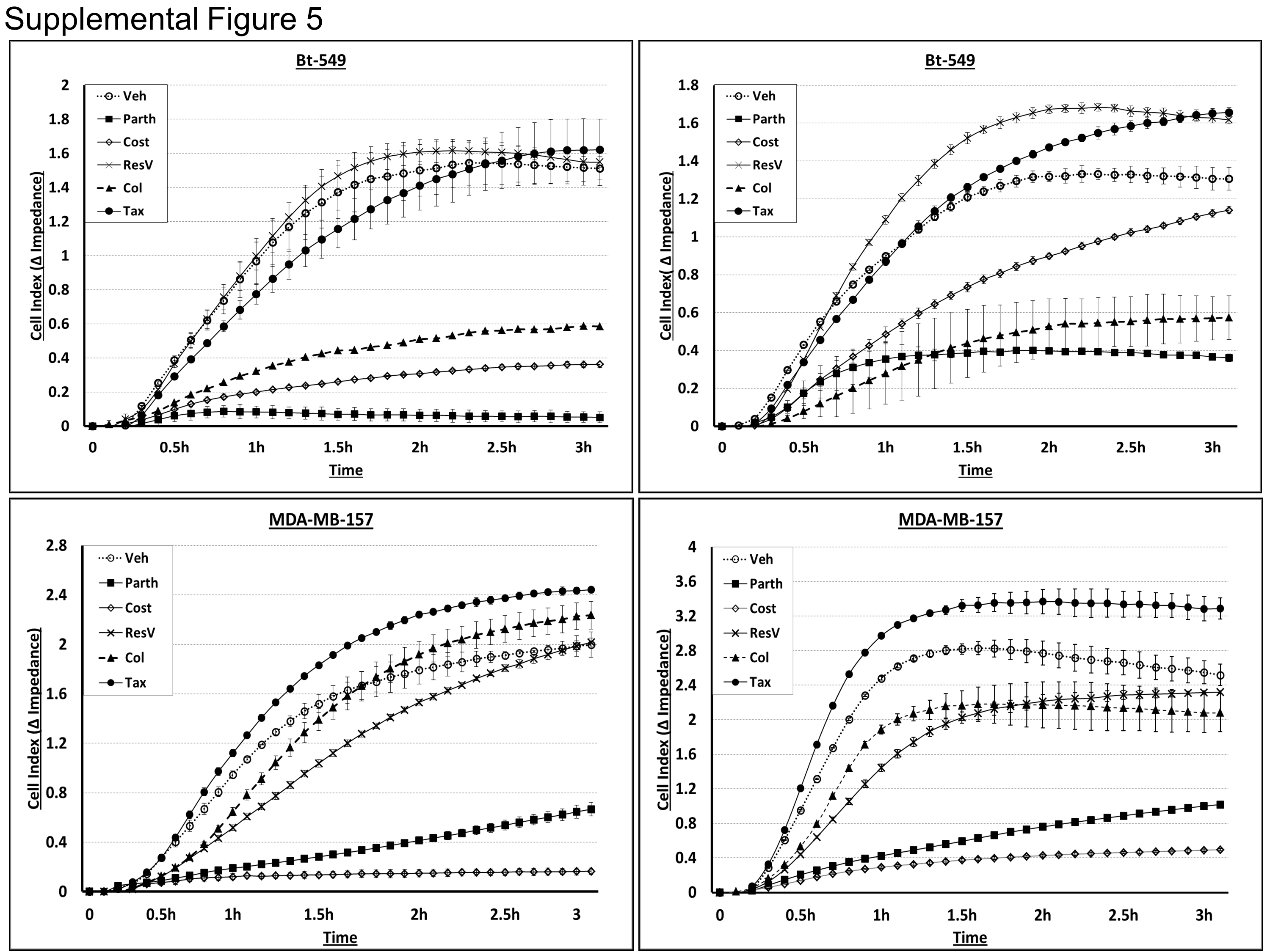

Supplement: Additional file 5: Figure S5 — Additional real-time electrical impedance monitoring experiment trials of Bt-549 and MDA-MB-157 showing that parthenolide (10 μM) and costunolide (25 μM) significantly reduce attachment when compared to vehicle while Taxol (0.5 μg/ml) and resveratrol (50 μg/ml) did not. Colchicine (50 μM) reduced attachment to a greater extent in Bt-549 than in MDA-MB-157. Lines, mean for three triplicate wells; bars, SD. [file bcr3477-S5.tiff]
